# Single video games improve cognitive functioning in college students: evidence from behavioral and fNIRS assessments

**DOI:** 10.3389/fpsyt.2025.1640142

**Published:** 2025-09-24

**Authors:** Chuangtao Li, Xiaodan Guo, Jingsong Wang, Shen Wang

**Affiliations:** ^1^ School of Physical Education and Sport Science, Fujian Normal University, Fuzhou, China; ^2^ Gdansk University of Physical Education and Sport, Gdańsk, Poland

**Keywords:** cognitive function, single video game, college students, behavioral response, fNIRS

## Abstract

**Objective:**

In the digital intelligence era, video games have become highly popular among college students, with the duration of playtime escalating rapidly. There is a growing research interest in video games to improve cognitive function, and video games have shown great potential in improving cognitive function. However, most of the current studies have focused on the effects of long-term gaming experience or short-period gaming training on cognitive functioning, and it remains unknown whether a single session of video gaming is equally effective. The primary aim of this study was to evaluate the impact of a single gaming session on the cognitive functions of college students and to explore its underlying mechanisms.

**Methods:**

Forty-three college students from a university in Fuzhou City were recruited and randomly assigned to either the VG group (video game) or the nVG group (non-video game). Pre- and post-test behavioral and functional near-infrared spectroscopy (fNIRS) data were collected from the participants. The statistics were analyzed using repeated measures ANOVA, with simple effects analysis conducted if interaction effects were significant, and corrections applied using the Bonferroni method.

**Results:**

(1) Following the video game session, the VG group exhibited shorter RT (reaction times), higher ACC (accuracy), and greater RCS (response correctness scores), whereas the nVG group experienced longer RT, lower ACC, and lower RCS. (2) In the VG group, post-test concentrations of Oxy-Hb in channels 6, 9, and 29 were elevated, particularly in the bilateral orbitofrontal cortex (OFC) and left dorsolateral prefrontal cortex (DLPFC), while there was minimal change in prefrontal cortex (PFC) activation levels in the nVG group.

**Conclusions:**

Cognitively engaging video games can effectively enhance the cognitive abilities of male college students. The underlying mechanism may be closely related to the promotion of prefrontal lobe activation by video games, which in turn improves reflective ability, processing speed, and decision-making levels.

## 1 Introduction

Cognitive function is an indispensable psychological foundation for college students in learning, socializing and daily life. League of Legends (LOL), as the most active Multiplayer Online Battle Arena (MOBA) game, is highly interactive and participatory (1). Secondly, the complexity of gameplay also requires players to possess advanced cognitive abilities such as visual selectivity, multi-target tracking skills, and executive functions (2–4), thus gradually becoming a new experimental paradigm to study cognitive neuroscience (5). Currently, it has been shown that video game training improves cognitive skills such as visual attention and information processing speed and demonstrates significant advantages in visual-spatial attention and information filtering (6). In addition, video game-induced plastic changes in brain function, covering reorganization at the prefrontal, parietal, hippocampal, and even brain network levels, similarly supporting its potential to improve cognitive function (7).

Functional near-infrared spectroscopy (fNIRS), as a non-invasive and portable brain functional imaging modality, can measure the changes in the concentration of oxyhemoglobin (Oxy-Hb) and deoxyhemoglobin (Deoxy-Hb) in the prefrontal cortex (PFC) and is one of the ideal tools for assessing the One of the ideal tools for assessing cognitive load and brain activation (8). It has been widely used to assess changes in prefrontal activity during cognitive tasks and has been gradually applied to the study of brain mechanisms in gaming tasks. Many previous neuroimaging studies have demonstrated that video game playing induces functional and structural plasticity in the PFC. In an adult study, self-reported playing time was correlated with cortical thickness in the left dorsolateral prefrontal cortex (DLPFC) (9) and microstructural properties of white matter in the PFC (10). However, the brain plasticity changes associated with video gaming and the brain mechanisms behind them remain unclear, which may be related to the sustained/burst activation or inhibition of the affected brain regions during gaming.

Although studies have investigated the cognitive effects of long-term video game training interventions (11), the immediate, acute effects of a single game session are relatively unexplored, and the neurocognitive mechanisms that underline the explanation of long-term adaptive change are incompletely understood (6). The advantage of a single video game session is that it allows us to isolate the purely cognitive “state” changes induced by the game itself from confounding variables such as long-term learning and structural brain changes (12). Furthermore, from a neurochemical perspective, a Positron Emission Tomography (PET) study has shown that video game experience triggers an immediate release of striatal dopamine (DA) (13). This dopamine release is a typical “state-specific” neuromodulator mechanism, with time constants that are highly consistent with the length of a single gaming session, rather than “idiosyncratic” changes spanning weeks or months.

The effects of a single video game session have been explored from a cognitive perspective, and one study (14) showed that a one-time-mobile action video game could selectively improve the efficiency of college students’ alerting network, suggesting that even a short period of time may trigger an immediate improvement in cognitive processing. However, the immediate effects of one-shot video games on the brain mechanisms of college students’ cognitive functioning have been less explored. Only a few longitudinal studies (15–18) found that weeks to months of video game training altered functional activity, resting-state functional connectivity (FC), and gray matter (GM) volume/cortical thickness in the DLPFC, as well as other brain regions such as orbital frontal cortex (OFC) (19) and striatum (16, 20). Whether a single video game session can alter our behavioral responses as well as activate the PFC remains well worth exploring.

In summary, the current literature has not systematically discussed whether a single 1h video game session can instantly enhance cognitive functioning in the college student population, not to mention the lack of empirical studies that combine synchronized recording of behavioral metrics (RT, ACC) with fNIRS metrics (Oxy-Hb). This study of a single video game session, the idea of combining behavioral and brain activation, fills the gap of the immediate cognitive effect of a single video game. It also proposes new ideas for stress reduction and learning efficiency improvement in higher education and triggers a re-evaluation of games as a cognitive training tool. The hypotheses of the study cover two aspects: (1) In terms of behavior, the VG group showed shorter RT, higher ACC, and greater RCS in the posttest, whereas the nVG group showed an increase in RT, a decrease in ACC, and a decrease in RCS in the posttest. (2) In terms of brain activation, the VG group showed increased PFC activation in the posttest compared to the pretest and exhibited higher levels than the nVG group.

## 2 Materials and methods

### 2.1 Participants

**Figure 1:**
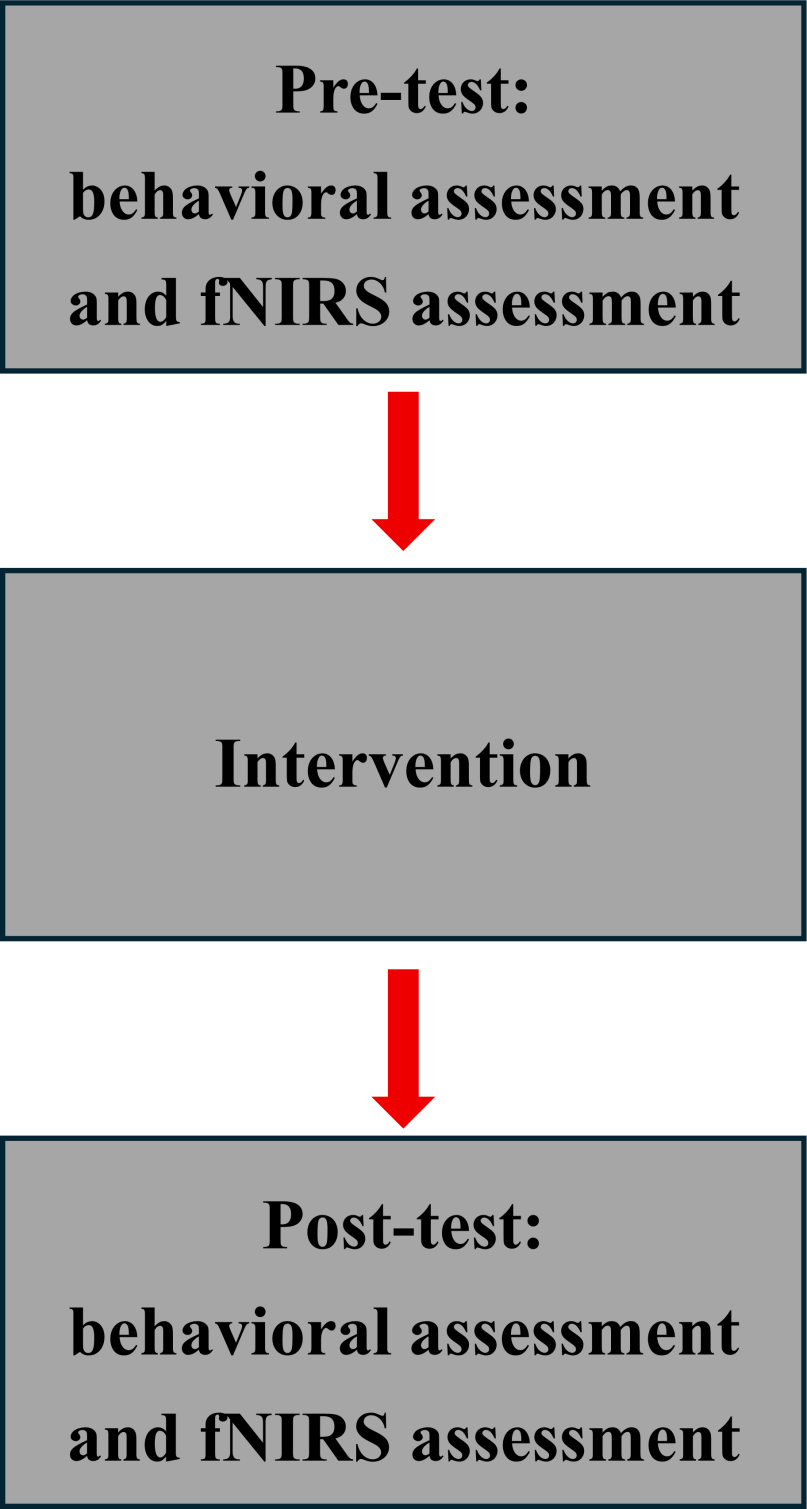
Experimental design.

This study was conducted from September 2024 to December 2024. In September 2024, the authors conceptualized this study to parallel subjects. Subjects were recruited mainly in Fuzhou City, China. *A priori* effects analysis was first performed using G*Power 3.1, with an effect size of 0.25 according to a previous study (21), a two-sided test was set with *α* = 0.05, and statistical efficacy was set at 0.80 (*β* = 0.20), and according to a 2-group, pre- and post-test (*df* = 2) repeated measures ANOVA, the sample size was 34, and considering subject compliance, a 20% expansion of the sample size was needed to require at least 41 people, and finally 43 people were actually tested and randomly assigned to the VG group (21.0 ± 1.9y, 177.0 ± 4.6cm, 67.7 ± 8.0kg) and nVG group (20.6 ± 1.6y, 177.5 ± 6.0cm, 70.1 ± 7.3kg). Inclusion criteria: (i) Male college students (22); (ii) Played LOL continuously for at least the past 6 months and >5h per week (23); (iii) Have an official ranking of no less than Gold in the season prior to the experiment (24); (iv) Normal visual acuity or corrected visual acuity; (v) Are right-handed; (vi) No consumption of stimulants, such as alcohol, tea, and coffee, before the experiment. Exclusion criteria: (i) Suffering from any disease affecting the central nervous system; (ii) Presence of cognitive dysfunction; and (iii) Ongoing participation in similar screen exposure studies. Subjects were informed of the overall experimental procedure (**Figure 1**), and after they agreed and signed the informed consent form, they would be paid $50 (RMB) for successful completion of the experiment. The study was conducted in strict accordance with the ethics of human research as stipulated in the Declaration of Helsinki and was approved by the Ethics Committee of Fujian Normal University (FNV-2025003).

### 2.2 Stimulus and procedure

#### 2.2.1 Stimulus preparation

The experiment consisted of three consecutive phases: pre-test on the cognitive functioning, a 1h game session, and post-test on the cognitive functioning (**Figure 1A**). The experimental paradigm in this study was a picture task. Eighty pictures of each of the three potencies: positive, neutral and negative were selected from the Chinese Affective Picture System (CAPS) (25). The validity and arousal level of the pictures were 9 levels, with 1 being the lowest validity and arousal level, and 9 being the highest validity and arousal level. The validity of the selected pictures was 7.1 ± 0.2 for positive, 5.4 ± 0.5 for neutral, and 2.5 ± 0.2 for negative. One-way ANOVA on the validity of the pictures in the three groups revealed that the validity of the pictures in the three groups was positive>neutral>negative, and the difference was significant (*F*_2,237_ = 3485.6, *P*<0.01); arousal levels were 5.4 ± 0.7 for positive, 5.0 ± 0.5 for neutral, and negative 5.1 ± 0.4.A one-way ANOVA analysis of the arousal of the three groups of pictures revealed that the difference in arousal of the three groups was not significant *F*_2,237_ = 181.4, *P*>0.05.

E-Prime 2.0 software was used to create picture tasks to measure cognitive functions. The picture task was designed in a block design with 5 blocks and 48 trials per block. All pictures had uniform settings for brightness, contrast, and saturation with 433 × 328 pixels. stimuli were presented with a Lenovo 15.6-inch laptop computer with a screen resolution of 1024 × 768 dpi and a black background. Subjects sat still in front of the computer screen, keeping their eyes at a horizontal distance of 50–70 cm from the screen.

#### 2.2.2 Overall procedure

##### 2.2.2.1 Pre-test on the cognitive functioning

Prior to the video game session, participants completed an assessment of cognitive functioning, consisting primarily of a behavioral assessment and an fNIRS assessment, both administered simultaneously. To minimize interference from external factors, participants sat in a dimly lit room that was soundproofed and had an electrically shielded test chamber. Participants were required to strictly follow the operator’s instructions throughout the experiment.

##### 2.2.2.2 Intervention

Subjects in the VG group arrived at the laboratory to receive a brief tutorial on the computerized LOL game. Prior to the start of the experiment, subjects performed a 10 minutes standardized practice session using the LOL program that had been pre-installed by the primary subject to avoid compromising data accuracy due to unfamiliarity with the equipment. Then it officially began with subjects playing the LOL game on the computer for 1 hour at full attention. The duration of a single intervention in this study was determined based on protracted battles in LOL and with reference to previous studies (23). “Victory” was defined as the side judged by the system to have won at the end of the game time, and “defeat” as the side judged by the system to have lost. To control the variable of character familiarity, all participants used a new character that they had never encountered before the experiment to eliminate the potential effect of familiarity differences. In addition, this study strictly controlled the duration of the game, with the main participant strictly timing the game and reminding the participants to stop the game immediately at the 60 minutes mark; subjects who played for less than 60 minutes were excluded from the study to avoid errors caused by the lack of time as much as possible. The final analysis is based only on games completed within the agreed time frame with a clear win/loss outcome.

Subjects in the nVG group watched 1h of LOL gameplay videos using another computer in the same experimental setting. With the primary subject selecting the video in advance and placing it on the desktop for backup. The purpose of selecting the same game for both groups was to ensure that the two groups were consistent in terms of visual/auditory stimuli, task semantics, event pacing, and screen exposure time, with the only systematic difference being whether real-time manipulation and decision-making (cognitive engagement) was performed. Reducing the effects of content differences, topic preference, and screen time allowed for a more accurate examination of the immediate effects of a single video game session on college students’ cognitive functioning.

##### 2.2.2.3 Post-test on the cognitive functioning

After the video game session, the participants completed another assessment of cognitive functioning. The exact procedure was identical to the pre-test of cognitive function. Each cognitive function assessment lasted approximately 12 min. There was a 10 min break in both the pre-video game cognitive function assessment and after the video game session. The entire experiment lasted approximately 100 min.

#### 2.2.3 Assessment

##### 2.2.3.1 Behavioral assessment

**Figure 2:**
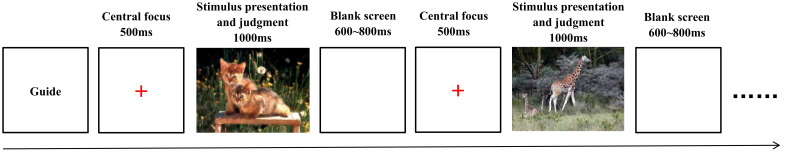
Schematic representation of the stimulus and experimental paradigm for cognitive function tests for college students. Source: Photographs from the Chinese Mood Picture System.

This section focuses on collecting behavioral data from the subjects, including response time and accuracy. The picture task was performed as follows, the instruction was presented first, and subjects pressed the space bar to initiate the experiment. A 500-ms “plus” gaze point appeared on the screen, followed by the presentation of the picture after the gaze point disappeared (at 1000 ms). Subjects were instructed to press a key to judge the picture immediately upon presentation (“D” for positive, “F” for neutral, “J” for negative). If there was no timely response, the data from that trial were not included in subsequent analysis, and the gaze point reappeared to signal the start of the next trial. The interval between trials was approximately 600–800 ms (an empty screen), and the prompt “D” appeared at the end of each trial, indicating a positive response, “F” for neutral, and “J” for negative. At the end of each block, the prompt “Please rest for 15 seconds” was displayed. At the conclusion of the entire experiment, an end message was shown on the screen. Prior to the experiment, a 2-minute practice session was conducted, followed by the formal experiment (**Figure 2**).

##### 2.2.3.2 fNIRS Assessment

**Figure 3:**
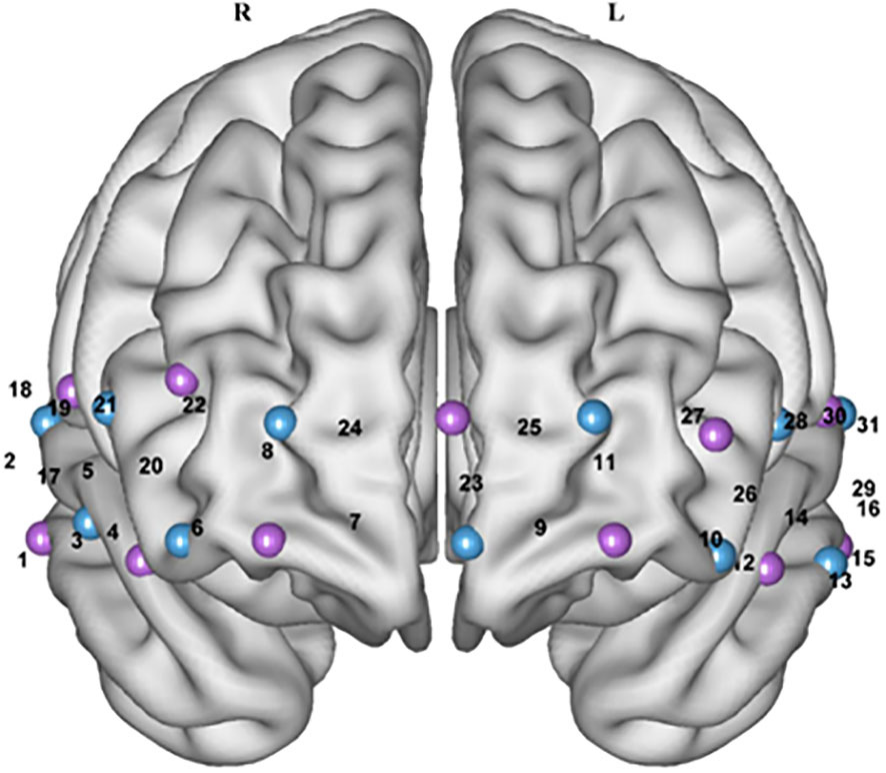
Schematic layout of fNIRS channels in the prefrontal brain region of the brain (Front view). Purple represents the light source, blue represents the probe, and the number represents the measurement channel position.

The fNIRS was used to test the concentration of oxygenated hemoglobin (Oxy-Hb), deoxy-hemoglobin (Deoxy-Hb) and total hemoglobin (Total-Hb) in the Participants. The system is a continuous-wave NIR system consisting of 11 light sources (the sources emit light at two wavelengths, 760 nm and 850 nm, with a sampling frequency of 11 Hz), and 11 probes, placed alternately at 3 cm spacing on a 2 × 11 grid to form 31 channels to cover the PFC (**Figure 3**).

Photopolar cap localization was referenced to the 10–20 International Electroencephalographic Localization System (26), combining the International Brain Region Probability Distribution (27) with the fNIRS photo polar localization criterion (28) as a means of obtaining the correspondence between all channels and Brudermann’s subdivisions (**Table 1**). Related studies (29) have found a relationship between the location of fNIRS channels and specific brain regions.

**Table 1:** The corresponding relationship between channel and PFC brain regions.

Brain regions	Channels
Frontopolar area, FPA	8、11、23、24、25
Orbitofrontal area, OFC	6、7、9、10
Dorsomedial prefrontal cortex, DMPFC	20、21、22、26、27、28
Ventromedial prefrontal cortex, VMPFC	3、4、5、12、13、14
Dorsolateral prefrontal cortex, DLPFC	17、18、19、29、30、31
Ventrolateral prefrontal cortex, VLPFC	1、2、15、16

### 2.3 Synchronous recording method

A local area network (LAN) was used to connect E-Prime and fNIRS for synchronized recording. Computer A was responsible for presenting stimuli and collecting behavioral data, while Computer B was responsible for collecting brain activation data. Upon presentation of the stimulus or when the subject made a response, Computer A transmitted the signal, and both computers simultaneously recorded the data and generated accurate timestamps to match the offline data.

### 2.4 Data processing and statistical analysis

#### 2.4.1 Behavioral data preprocessing

Behavioral data were organized using E-data. Reaction time data were only counted for trials with correct responses. To reduce the influence of extreme values, referring to the previous study (30), trial times with reaction times were less than 150ms or more than 1500ms and outside the range of mean ± 3 standard deviations were excluded. Meanwhile, to compensate for the shortcomings of a single index, the RCS was added as a response speed index for correct response adjustment. The RCS is calculated as the number of correct responses divided by the total response time, reflecting the number of correct responses per unit of time. A larger RCS indicates better cognitive function performance (31).

#### 2.4.2 fNIRS data preprocessing

The fNIRS data were preprocessed using NirSpark V1.5.20 (Jiangsu Danyang Huichuang Medical Equipment Co., Ltd.). The raw optical density data were corrected for motion artifacts using a wavelet-based motion de-artifacting method (32). The data were then band-passed using a filter of 0.2-0.01 Hz. Based on the modified Beers-Lambert law, the filtered optical density data were converted to Oxy-Hb, Deoxy-Hb, and Total-Hb concentrations. Since the Oxy-Hb signal is more sensitive in measuring cerebral blood flow and has a better signal-to-noise ratio and re-measurement reliability, which can truly reflect the level of neural activation in the brain (33), Oxy-Hb data were used in all subsequent analyses. After obtaining the raw Oxy-Hb data of the 31 channels, the mean value of Oxy-Hb under all trials was calculated, and the average value of each sampling point per unit time for each channel of the subjects could be obtained.

#### 2.4.3 Statistical analysis

Preliminary descriptive statistics were calculated; measurements are expressed as ( $\bar{x}\pm s$ ) and count as [n (%)]. A preliminary assessment of outliers was conducted using Q-Q plots, and no significant bias was found. Normality and homogeneity of the samples were confirmed using the Shapiro-Wilk test and Levene’s test, respectively, which indicated normality (*P*>0.05) and homogeneity (*P*>0.05) within the samples. Repeated measures ANOVA were then conducted, including time (pre-test, post-test) × group (VG, nVG) to explore potentially significant interactions. Simple effects were examined if the interaction was significant and corrected using the Bonferroni method. Cohen’s “small/medium/large” thresholds (e.g., $\eta_{P}^{2}$ ≈0.01/0.06/0.14) have traditionally been used for qualitative interpretation (34). Statistical analyses were carried out using SPSS (IBM Corp. Released 2021. IBM SPSS Statistics for Windows, Version 28.0. Armonk, NY: IBM Corp.), with statistical significance set at *p*<0.05.

## 3 Results

### 3.1 Behavioral results

**Figure 4:**
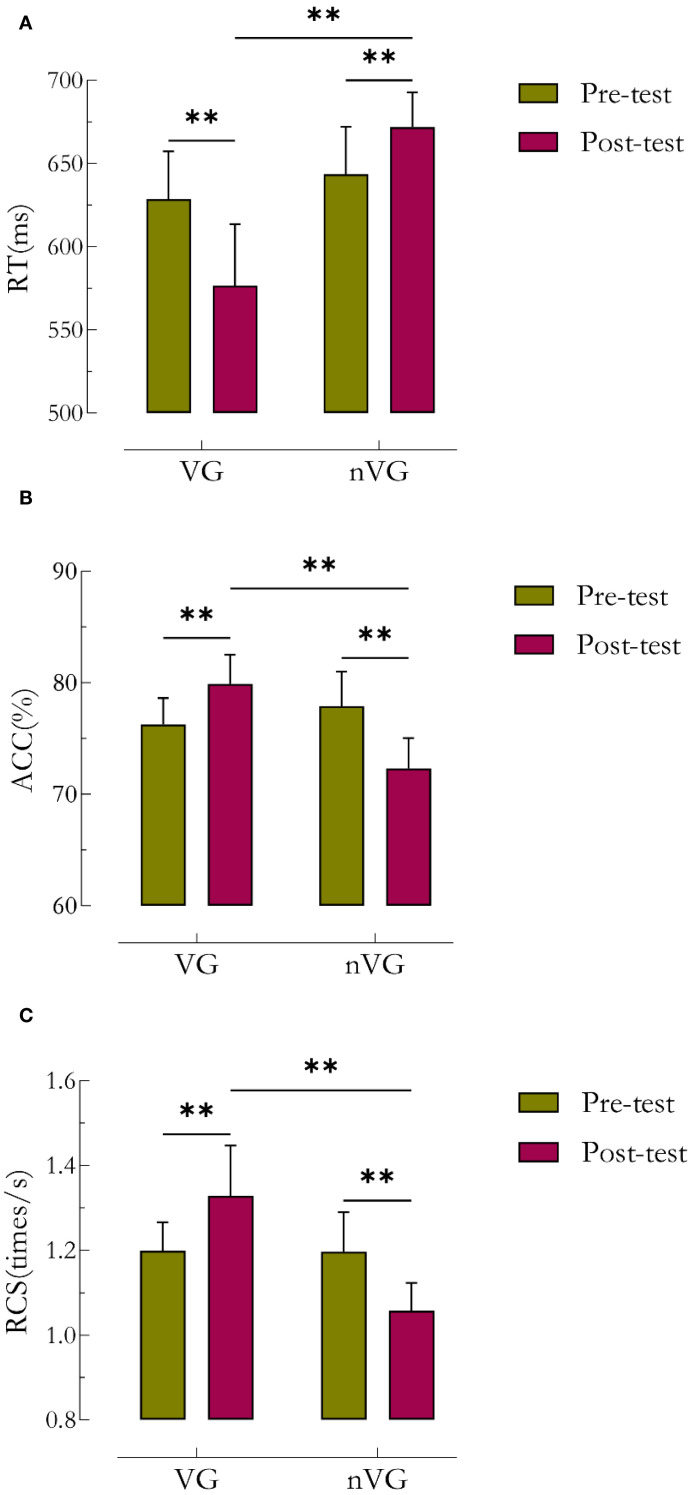
Comparative analysis of behavioral outcomes before and after video game intervention in the VG and nVG groups. ^a^ denotes RT, ^b^ denotes ACC, and ^c^ denotes RCS. ^*^ denotes *P*<0.05, ^**^ denotes *P*<0.01. RCS is an index of response speed adjusted for correct responses, and RCS = number of correct responses/total response time.

**Table 2** and **Figure 4** illustrate the statistical results of the behavioral pre- and post-tests. The repeated-measures ANOVA results indicated a significant main effect of the RT group (*F*_1,41_ = 45.6, *P*<0.01, $\eta_{P}^{2}$ =0.5), a significant main effect of time (*F*_1,41_ = 9.9, *P*<0.01, $\eta_{P}^{2}$ =0.2), and a significant group × time interaction effect (*F*_1,41_ = 114.5, *P*<0.01, partial $\eta_{P}^{2}$ =0.7). The group main effect, time main effect, and group × time interaction effect of RT with $\eta_{P}^{2}$ greater than 0.14, indicating a large effect size. Further simple effect analyses revealed that the posttest RT was significantly lower for VG (*P*<0.01) with a difference of 52.0ms (95% CI: 2.38 ~ 3.59), and significantly higher for nVG (*P*<0.01) with a difference of 28.4ms (95% CI: -2.25 ~ -1.01). There was a significant difference in posttest RT between the two groups, with a difference of 95.4ms (95% CI: -6.52 ~ -4.45).

**Table 2:** Descriptive statistics of behavioral pre- and post-tests.

Group	Times	*N*	RT (ms)	ACC (%)	RCS (times/s)
VG	Pre-test	22	628.5 ± 28.8	76.3	1.2 ± 0.1
	Post-test	22	576.5 ± 37.1	79.9	1.3 ± 0.1
nVG	Pre-test	21	643.6 ± 28.5	77.9	1.2 ± 0.1
	Post-test	21	672.0 ± 20.9^ab^	72.3^ab^	1.1 ± 0.1^ab^
Group			45.6**	15.9**	34.6**
Time			9.9**	9.9**	0.1
Group × Time			114.5**	166.4**	89.2**

a *P*<0.01 vs VG group; *P*<0.01 vs pre-test; *P<0.05, **P<0.01.

There was also a significant main effect of the ACC group (*F*_1,41_ = 15.9, *P*<0.01, $\eta_{P}^{2}$ =0.3), a significant main effect of time (*F*_1,41_ = 9.9, *P*<0.01, $\eta_{P}^{2}$ =0.2), and a significant group × time interaction effect (*F*_1,41_ = 166.4, *P*<0.01, $\eta_{P}^{2}$ =0.8). The group main effect, time main effect, and group × time interaction effect of ACC with $\eta_{P}^{2}$ greater than 0.14, indicating a large effect size. Further simple effect analyses showed that the posttest ACC was significantly higher for VG (*P*<0.01) with a difference of 3.64% (95% CI: -2.80 ~ -1.60), and significantly lower for nVG (*P*<0.01) with a difference of 5.56% (95% CI: 2.75 ~ 3.98). There was a significant difference in posttest ACC between the two groups, with a difference of 7.57% (95% CI: 3.58 ~ -5.58).

Lastly, there was a significant main effect of the RCS group (*F*_1,41_ = 34.6, *P*<0.01, $\eta_{P}^{2}$ =0.5), no significant main effect of time (*F*_1,41_ = 0.1, *P*>0.05, $\eta_{P}^{2}$ =0.0), and a significant group × time interaction effect (*F*_1,41_ = 89.2, *P*<0.01, partial $\eta_{P}^{2}$ =0.7). The time main effect of RCS was 0.003, indicating a small effect size; the $\eta_{P}^{2}$ for both the group main effect and the group × time interaction effect was greater than 0.14, which is a large effect size. Further simple effect analyses indicated that the posttest RCS was significantly higher for VG (*P*<0.01) with a difference of 0.13 times/s (95% CI: -2.57 ~ -1.36), and significantly lower for nVG (*P*<0.01) with a difference of 0.14 times/s (95% CI: 1.49 ~ 2.72). There was a significant difference in posttest RCS between the two groups, with a difference of 0.27 times/s (95% CI: 3.28 ~ 4.93).

### 3.2 fNIRS results

**Figure 5:**
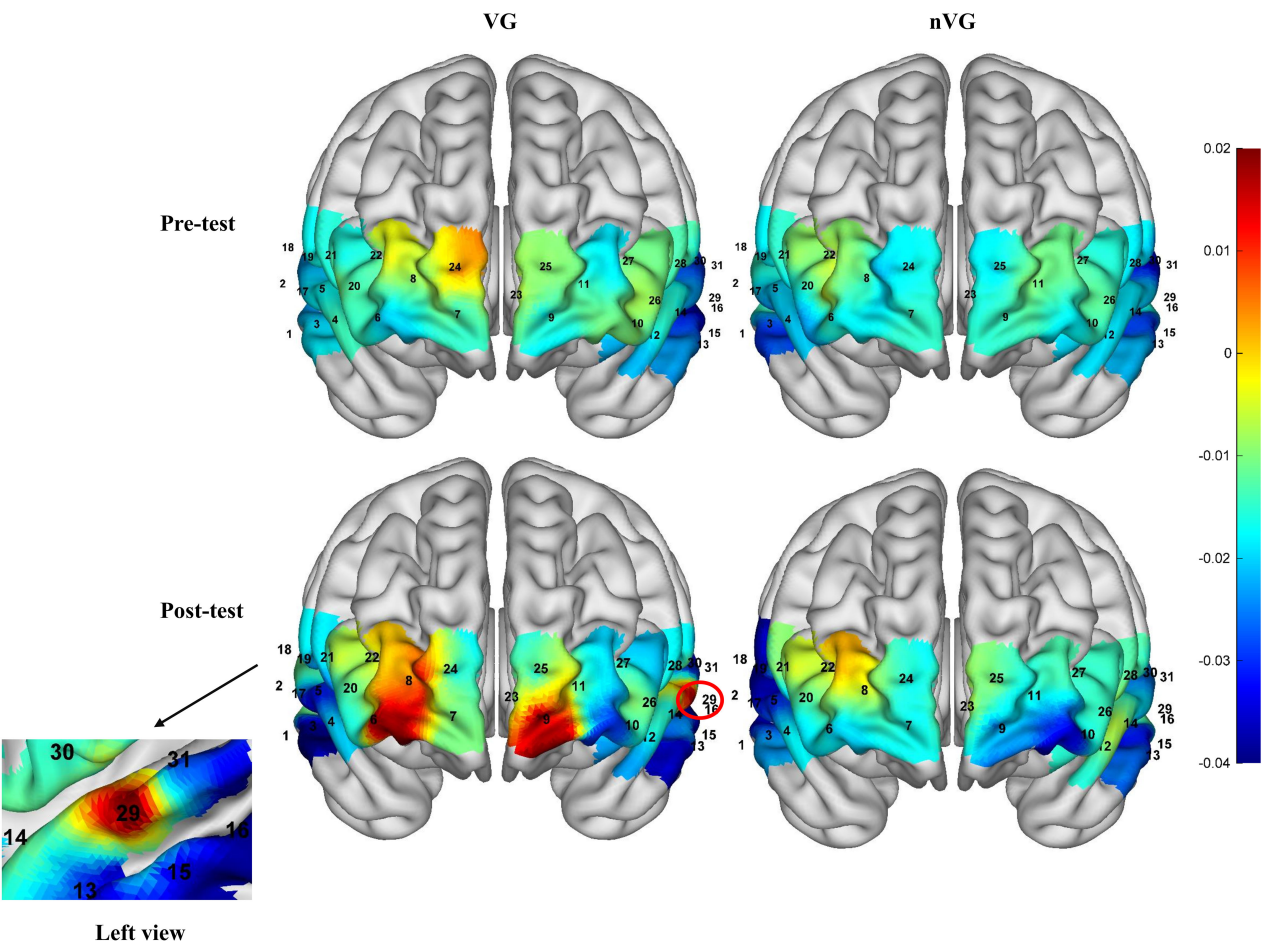
Schematic representation of activation in prefrontal brain regions of the brain before and after video game intervention in the VG and nVG groups.

**Figure 5** illustrates the activation of the PFC during pre- and post-measurements of VG and nVG. A repeated-measures ANOVA analysis of the mean Oxy-Hb concentration in each channel revealed that There were no significant differences (*P*>0.05) in the time main effect, main category main effect, and group × time interaction effect for each of the 3 channels, including channel 6, channel 9, and channel 29; yet channels 6 (*F*_1,41_ = 5.0, *P*<0.05, $\eta_{P}^{2}$ =0.1), 9 (*F*_1,41_ = 5.9, *P*<0.05, $\eta_{P}^{2}$ =0.1), and 29 (*F*_1,41_ = 4.2, *P*<0.05, $\eta_{P}^{2}$ =0.1) exhibited significant group × time interaction effects. And the $\eta_{P}^{2}$ for the group × time interaction effect for the three channels, channel 6, channel 9, and channel 29, all ranged from 0.06 to 0.14, indicating a medium effect size.

**Figure 6:**
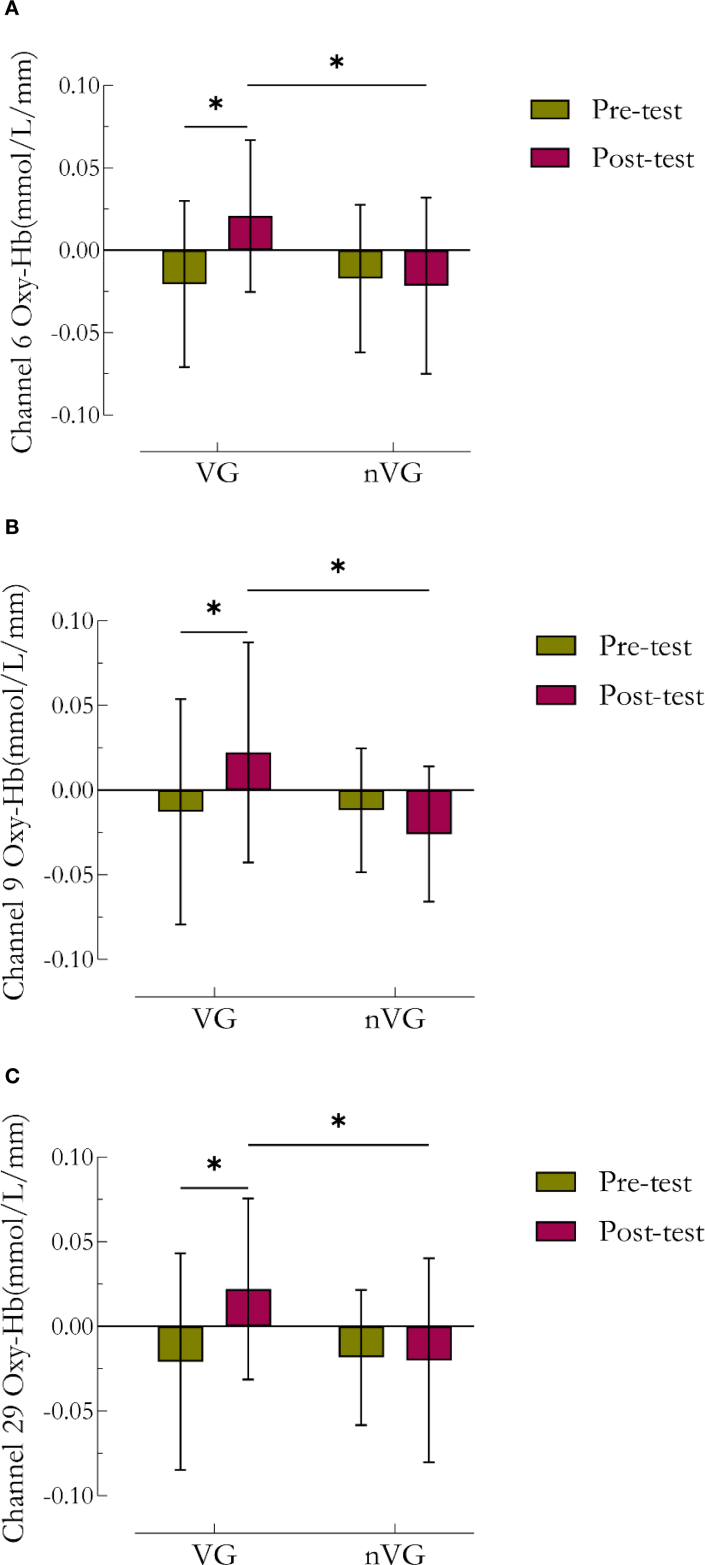
Comparative analysis of channel 6, channel 9, and channel 29 Oxy-Hb concentration results before and after video game intervention in the VG and nVG groups.^a^ denotes channel 6, ^b^ denotes channel 9, and ^c^ denotes channel 29. ^*^ denotes *P*<0.05, ^**^ denotes *P*<0.01.

Bonferroni multiple comparisons of time under different group conditions indicated that in channels 6 (**Figure 6A**), 9 (**Figure 6B**), and 29 (**Figure 6C**), the concentrations of Oxy-Hb were significantly higher in the VGP posttests compared to the pretests (channel 6: *t*=-2.90, *P*<0.05, Cohen’s *d*=-0.87; channel 9: *t*=-2.36, *P*<0.05, Cohen’s *d*=-0.75; channel 29: *t*=-2.78, *P*<0.05, Cohen’s *d*=-0.85). Furthermore, Bonferroni multiple comparisons of groups at different time conditions revealed that at the pre-test, the Oxy-Hb signals in channels 6, 9, and 29 did not significantly differ between the two groups (*P*>0.05); however, at the post-test, the Oxy-Hb concentrations in channels 6, 9, and 29 of the nVGP were significantly lower than those of the VGP (channel 6: *t* = 2.83, *P*<0.05, Cohen’s *d* = 0.89; channel 9: *t* = 2.92, *P*<0.05, Cohen’s *d* = 1.03; channel 29: *t* = 2.42, *P*<0.05, Cohen’s *d* = 0.83).

In summary, based on the brain regions corresponding to each channel, the prefrontal regions with significant changes were the bilateral OFC and the left DLPFC.

## 4 Discussion

This study examined whether plasticity in cognitive function could be observed after only a 1h VG session only. Forty-three healthy current male college students were recruited, randomly assigned to the VG and nVG groups, and asked to complete assessments of cognitive functioning before and after a 1h VG session. The study used both behavioral and fNIRS measures, the latter for its high temporal and spatial resolution. Within-subjects analyses compared participants’ performance before and after the session. The results showed that the VG group had shorter RT, higher ACC, and higher RCS, while the nVG group had longer RT, lower ACC, and lower RCS after the test. There were significant differences in behavioral performance between the two groups. Then, compared with the nVG group, the VG group exhibited significantly higher Oxy-Hb concentrations in bilateral OFC and left DLPFC after the video game task, while the activation level of PFC in the nVG group remained almost unchanged. These results effectively validated our previous hypothesis. The PFC is mainly responsible for the regulation of higher cognitive functions, and its structural and functional complexity makes it a core brain region for human cognition, decision-making, emotion regulation and social behavior (35). This cortical region is ideally positioned to regulate cognitively relevant responses in a changing environment.

As shown by our findings, after a brief video game training, the VG group improved cognitive reaction speed, and on the contrary, the nVG group showed a significant decrease in reaction speed, which is in line with the results of previous studies (36). A common advantage embodied by video games is the shortening of reaction time (33, 37, 38). Dye combed through the literature related to video games and reaction time and found that video games were not only associated with faster reaction times, but also observed a significant linear relationship between VG and nVG reaction times in task and task conditions, and that VGs were about 10-12% faster than nVGs, even though they used different study tasks (visual search, N-back working memory, Go/No-go, etc.), the overall reaction times differed by almost an order of magnitude, i.e., from a task with a very fast average reaction time (about 200ms) to a task with a rather slow average reaction time (about 2000ms), and this relationship still existed (39). Second, this study found that the ACC and RCS of the VG group were also effectively improved after the video game. This may be because video games require players to track multiple dynamic targets (e.g., enemies, props) at the same time, and this training enhances visuospatial attentional breadth and selective attentional abilities. Research from Nottingham Trent University in the UK noted that those who played games 1-5h a week were 5% more accurate in processing virtual information and had brain scans that showed enhanced activity in areas such as the lingual gyrus, supplementary motor area, and thalamus, compared to those who never played games (40). This finding is supported by research from the University of Oxford, which suggests that gaming leads to positive emotional experiences through “anticipatory effects”(e.g., reward mechanisms), which in turn enhance cognitive efficiency (41).

Furthermore, the association between video gaming and cognitive enhancement and PFC activation has been confirmed by experts (9, 42, 43). Compared to non-video gamers, video gamers show more integrated white matter in motor and visual pathways (44) and higher levels of activation in PFC brain regions, despite comparable cognitive performance (43). This was also demonstrated in the present study. In addition, it has also been shown ^[9.43]^ that playing video games for longer periods of time is also associated with cortical thickening in brain regions related to attention, navigation, visuomotor function, and ambiguity resolution (left orbitofrontal region, left dorsolateral prefrontal cortex (DLPFC), and bilateral internal olfactory cortex). That is, although the correlation between video games and changes in brain regions is more centralized, playing video games has also been associated with cognitive enhancement. Green and Bavelier’s review of research on video games and individual cognitive abilities also found that most of the research supports that gaming experiences are associated with cognitive enhancement, covering low-level perceptual skills to high-level cognitive flexibility, including levels of visual decision-making, processing speed, task-switching ability, and the ability to perform multiple tasks simultaneously (45). One other study (4) recruited 29 healthy undergraduate and graduate students as subjects to explore whether cognitive and neural plasticity could be observed after a brief video game. The results found that visual selective attention was superior in the VG group to the nVG group after the game, suggesting that VSA plasticity is observable after 1 hour of video gaming. A single session of video gaming enhances prefrontal activation in the brain, and in terms of neurobiological mechanisms, subjects can easily trigger the brain’s reward system during video gaming, thus promoting dopamine release (46), and this dopamine surge not only reinforces gaming-related learning behaviors, but also modulates the arousal level of neurons within the PFC, thus optimizing higher-level working memory, attention allocation, and cognitive flexibility cognitive functions. Moreover, short windows of reward and arousal can increase the working points and neural gain of prefrontal networks, resulting in improved cognitive performance and enhanced prefrontal (DLPFC-containing) responses that can be observed in a single 1h paradigm (13). In the present study we observed an acute effect of neural arousal due to video games, while repeated participation in video game training may induce long-term synaptic plasticity, thus promoting enhanced synaptic connectivity and neural efficiency within the PFC.

Another noteworthy finding was that the nVG group showed a trend of decreasing cognitive benefit in the posttest. Although this finding was not the primary focus of this study, it still needs to be interpreted with caution. We speculate that it may be related to the type of activities that the two groups engaged in on-screen. LOL belongs to the Multiplayer Online Battle Arena (MOBA) games, where players are faced with dynamic and changing situations during gameplay, requiring no more than monitoring the battlefield, keeping track of multiple complex visual stimuli at the same time and responding to these stimuli under stringent time pressures (2). The complexity of the gameplay has a training effect on various cognitive abilities (visual processing, attention, multi-target tracking skills, and inhibitory control) of the players (3). Therefore, the VG group had cognitive, social, or physical involvement (playing video games, operating apps, etc.), which may be interactive, reciprocal, and mentally active, and is mentally active screen use (47). On the other hand, the nVG group watched LOL game videos for the same amount of time, and passively received information from the screen (watching swipe videos, watching TV), which is mentally passive screen use (47), more “stimulus-driven”, lacking goal orientation and action consequences, and is associated with over-activation of the default network, which decreases task-evoked responses and functional connectivity in the prefrontal control network, thereby reducing task-evoked responses and functional connectivity in the subsequent task-evoked response. This has been associated with over-activation of the default network, resulting in reduced task-evoked responses and functional connectivity in the prefrontal control network, and thus reduced performance on subsequent cognitive tests requiring executive control. Several studies have now proposed that there are differences between mentally active and mentally passive screen use in physical and mental illness (48), sleep difficulties (49), anxiety (50), and depression (50). So, are there differences in cognitive functioning between college students with mentally active and mentally passive screen use? This deserves further exploration.

Whereas prolonged video game playing did not lead to cognitive impairment, only a few reported cognitive impairments. Contrary to the negative media perception of video games and video game playing, game playing is associated with cognitive enhancement (43, 44). Well-controlled training studies have also shown a causal relationship between game playing and various enhancements, a fact that suggests that people who play games have better perceptual skills than those who do not. This work has led to an assessment of the practical applications of evaluating video game training, such as the potential tool that could be used as a gaming intervention for young people with psychological disorders (51). However, it is worth noting the potential risks associated with playing video games, where high-frequency action game players perform normally in response control tasks (e.g., stop-signal tasks) but have impaired prosodic control, as evidenced by significantly lower sequence effects than non-players. This difference may be related to the type of game - fast-response games reinforce immediate decision-making but impair anticipatory adjustment to complex background information. In addition, excessive gaming may lead to imbalances in prefrontal-limbic system regulation, manifested as decreased impulse control and impaired emotion regulation. In adolescent gaming addicts, over-responsiveness of the DLPFC to reward signals may exacerbate imbalances in cognitive resource allocation (52). Therefore, the relationship between playing video games and cognitive changes needs to be explored in a more objective manner.

Shortcomings and prospects: First, this study focused on the effect of VG on cognitive function in male college students but lacked gender comparison. In the future, female subjects need to be added to explore the relationship between VG and the improvement of cognitive function in the college student population. Second, fNIRS was selected to explore the effect of VG on PFC blood flow by noninvasive optical neuroimaging, but whether a single session of video gaming alters the structural changes within the brain needs to be explored in-depth with the help of fMRI technology. The results of this study are summarized in the following sections. Then, Although the use of an emotion picture task in the present study was able to increase the ecological validity of the experiment, it nevertheless failed to completely rule out confounding effects of emotion on task performance, and the results may reflect the joint role of cognitive functioning and emotion processing. Therefore, future research suggests the use of an experimental task that is emotionally neutral but matched for cognitive demands to more clearly separate cognitive functioning from emotional processing. Finally, although encouraging immediate effects were observed in this study, the long-term persistence of these benefits is unknown. The observed effects may be temporary and diminish over time. Future studies should incorporate delayed testing (e.g., after 1 hour, 24 hours, or 1 week) to explore the durability of cognitive enhancement and determine the optimal “dosage” to achieve sustained benefits.

## 5 Conclusion

Single video game can effectively promote the activation of PFC and improve cognitive function in male college students, and the mechanism may be closely related to the improvement of cognitive function by improved reactivity and increased Oxy-Hb concentration. This study provides a scientific basis for the use of video games in cognitive function interventions and theoretical support for the design of targeted video game intervention programs.

## Data Availability

The original contributions presented in the study are included in the article/supplementary material. Further inquiries can be directed to the corresponding author.
